# Beta Cell Count Instead of Beta Cell Mass to Assess and Localize Growth in Beta Cell Population following Pancreatic Duct Ligation in Mice

**DOI:** 10.1371/journal.pone.0043959

**Published:** 2012-08-30

**Authors:** Marie Chintinne, Geert Stangé, Bart Denys, Zhidong Ling, Peter In ‘t Veld, Daniel Pipeleers

**Affiliations:** 1 Diabetes Research Center, Brussels Free University-VUB, and Center for Beta Cell Therapy in Diabetes, Brussels, Belgium; 2 UZ Brussel, Department of Pathology, Brussels, Belgium; University of British Columbia, Canada

## Abstract

**Background:**

Pancreatic-tail duct ligation (PDL) in adult rodents has been reported to induce beta cell generation and increase beta cell mass but increases in beta cell number have not been demonstrated. This study examines whether PDL increases beta cell number and whether this is caused by neogenesis of small clusters and/or their growth to larger aggregates.

**Methodology:**

Total beta cell number and its distribution over small (<50 µm), medium, large (>100 µm) clusters was determined in pancreatic tails of 10-week-old mice, 2 weeks after PDL or sham.

**Principal findings:**

PDL increased total beta cell mass but not total beta cell number. It induced neogenesis of small beta cell clusters (2.2-fold higher number) which contained a higher percent proliferating beta cells (1.9% Ki67+cells) than sham tails (<0.2%); their higher beta cell number represented <5% of total beta cell number and was associated with a similar increase in alpha cell number. It is unknown whether the regenerative process is causally related to the inflammatory infiltration in PDL-tails. Human pancreases with inflammatory infiltration also exhibited activation of proliferation in small beta cell clusters.

**Conclusions/significance:**

The PDL model illustrates the advantage of direct beta cell counts over beta cell mass measurements when assessing and localizing beta cell regeneration in the pancreas. It demonstrates the ability of the adult mouse pancreas for neogenesis of small beta cell clusters with activated beta cell proliferation. Further studies should investigate conditions under which neoformed small beta cell clusters grow to larger aggregates and hence to higher total beta cell numbers.

## Introduction

Beta cell progenitor cells have recently been reported in the adult mouse pancreas [1] They were identified as ngn3-expressing cells that were induced by duct ligation, and that preceded an increase in beta cell mass. Although these cells were shown to differentiate to beta cells, it was not clear to which extent this process was responsible for an increase in beta cell number. The occurrence of beta cell neogenesis following pancreatic duct ligation (PDL) is still under debate, as is also its origin [2]–[10]. It has also been questioned whether beta cell mass correctly assesses beta cell numbers [8]. We have developed a method that directly counts beta cell numbers and that examines to which extent differences result from neoformation of small beta cell clusters and/or from their growth towards larger aggregates [11]. It was thus shown that 90 percent of beta cells in 10 week-old rats have been formed postnatally, involving massive neogenesis of small beta cell aggregates consisting of 1 to 13 cells and growth of a very small number of them to larger diameters, up to those of islets that are usually studied after isolation [11]. We have now used this method to investigate whether PDL in adult mice increases their total beta cell number, and, if so, whether this involves neoformation of small beta cell clusters and/or their growth to larger aggregates. PDL was not found to increase total beta cell numbers but induced the neoformation of small beta cell clusters with elevated levels of proliferating beta cells. This finding led us to investigate the small clusters in human donor organs that also contained inflammatory cell infiltrates, similar to those observed in the ligated mouse pancreas.

## Materials and Methods

### Animals

The study was conducted on 8 to 10-week old male BalbC mice (body weight 21.5 to 26.5) from three different shipments (one from Charles River Laboratories, Brussels, Belgium and two from Elevage Janvier, Le Genest-Saint-Isle, France). The duct of the pancreatic tail part was ligated as described by Xu et al [1]; for each set of duct-ligated animals an equal number from the same shipment was taken as sham operated (unligated) controls. Studies were approved by the Commissie Proefdiergebruik (CPG) of the Vrije Universiteit Brussel (VUB) (05/274/2) and conducted according the Principles of Laboratory Care, supervised by a qualified veterinarian.

### Tissue Processing

Two weeks after the intervention, animals were sacrificed by cervical dislocation and their pancreas was dissected. At the time of sacrifice, PDL and sham animals did not differ in 2-hour fasting and non-fasting glycemia or in glucose tolerance curves (data not shown). The tissue was cleared from lymph nodes and fat, separated into a duodenal part (‘head’) and a splenic part (‘tail’); in ligated pancreases, these parts were identified by the site of suture, while unligated tails were divided into head and tail portions at the corresponding site. The validity of this approach was supported by the identical weights of the head portions in ligated and unligated pancreases (Table 1). After weighing, head and tail parts were fixed overnight in formalin 4%, embedded in paraffin and entirely sectioned and sampled [11]. The beta cell populations were counted in systematically sampled sections that had been deparaffinised and stained for insulin (guinea pig anti-insulin, in-house, 1∶1000, overnight 4°C; secondary antibody: Alexa Fluor 647-conjugated anti-guinea pig from Invitrogen, Paisley, UK; 1∶500, 1 h room temperature); nuclei were stained with DAPI (Sigma-Aldrich, St Louis, MO, USA) in fluorescent mounting medium (Dako, Glostrup, Denmark). At this insulin antibody concentration, the contrast between insulin-positive cells and background was strongest (high signal over noise ratio), allowing high-throughput and specific image analysis. Insulin-positive cells were analysed for their Ki67-positivity in sections that had been pretreated for antigen retrieval (citrate buffer-pH 6.0 at 99°C) before overnight incubation at 4°C with rabbit anti-Ki67 (1∶50; Acris, Herford, Germany) and anti-insulin antibodies. Alpha cell populations were counted in sections stained for glucagon (rabbit anti-glucagon, in-house, 1∶1000, overnight 4°C; secondary antibody: Cy3-conjugated anti-rabbit from Jackson ImmunoResearch Laboratories, Suffolk, UK, 1∶500, 1 h room temperature) and DAPI. The inflammatory infiltrate was characterized with immunohistochemistry for CD45 (a broad leucocytic marker) in sections stained for CD45 (pretreatment citrate buffer-pH 6.0 at 99°C, rat anti-mouse CD45, 1∶25, BD Pharmingen, Erembodegem, Belgium).

**Table 1:** Effect of duct ligation on beta and alpha cell population in pancreatic tail.

	Shamn = 10	PDLn = 10
Body weight	24.3±1.4	24.2±1.7
Pancreas weight (mg)		
Tail	165±32	38±6***
Head	174±56	169±42
**Pancreas tail**		
Number of insulin-pos aggregates (10^3^)		
Total	2.3±0.5	4.7±2.0*
Per size category		
12–50 µm	1.9±0.4	4.2±1.9**
50–100 µm	0.22±0.05	0.28±0.07*
>100 µm	0.14±0.05	0.18±0.04
Number of beta cells (10^3^)		
Total	255±85	272±53
Per mg tissue	1.5±0.3	7.3±1.1***
Per g body weight	10.6±3.8	11.3±2.0
Per size category of aggregates		
12–50 µm	11±2	21±8**
50–100 µm	24±6	31±6*
>100 µm	220±79	221±44
Beta cell mass (mg)	0.96±0.33	1.53±0.41**
Beta cell volume (µl)	0.44±0.16	0.52±0.10
Percent insulin-positive area (of total)	0.56±0.12	4.11±0.99***
Beta cell properties		
Cell size (µm^2^)	171±19	185±11
Proliferative activity (Ki67+ cells)		
%	0.5±0.2	1.2±0.5**
Number (10^3^)	1.4±0.6	3.2±1.2***
Number of alpha cells (10^3^)		
Total	81±17	109±19**
Per mg tissue	0.5±0.1	3.0±0.6***
Per g body weight	3.4±0.9	4.6±1.0***
Per size category of aggregates		
12–50 µm	4±1	9±3***
50–100 µm	9±3	17±8**
>100 µm	68±17	83±18
Alpha cell mass (mg)	0.29±0.08	0.55±0.09***
Alpha cell volume (µl)	0.13±0.04	0.19±0.04*
Percent glucagon-positive area (of total)	0.18±0.04	1.50±0.34***

Data represent means ± SD.

Statistical analysis by Student’s *t* test.

* *p<*0.05.

** *p*<0.01.

*** *p*<0.001.

### Quantification of Beta Cell Number and Beta Cell Distribution Over Aggregates of Different Size

Minimally 3 percent of pancreatic tail portion was analyzed for determining beta cell counts with relative error under 10% [11]. For ligated tails, one section every 150 µm was sampled and the whole section digitally imaged using a Pathway 435 or 855 [11], covering a surface area of 0.9±0.05 cm^2^ (15±2 sections/tail). Unligated tails were sampled every 20–150 µm, for a total surface area of 9.2±2.1 cm^2^ (13±7 sections/tail).

Photographed sections were analyzed with imaging software (IPLab; Becton Dickinson, San Jose, CA, USA) to measure insulin-positive and insulin-negative surface areas, and to determine the number of nuclei in the insulin-positive areas. Total beta cell number is obtained from beta cell counts in sections after correction for cut nuclei [11]. Similarly, the number of beta cell aggregates comes from counts in sections following correction for cut surface assuming a spherical shape. Beta cell mass (mg per pancreas) was calculated by multiplying relative insulin-positive area (the percentage of insulin positive area over total pancreas area) by pancreas weight. Total beta cell volume (µl per pancreas) was determined by the Cavalieri principle: the insulin positive area was measured in each section and multiplied by the distance to the next section; the sum of these volumes gives the total volume [12], [13]. The average beta cell size was obtained by dividing beta cell volume by total beta cell number, and then calculating cross sectional area, assuming that the cells are spherical. Total alpha cell number, alpha cell mass (mg per pancreas) and alpha cell volume (µl per pancreas) were similarly determined in glucagon stained sections. Alpha cell numbers in sized aggregates were derived from their surface area relative to the beta cells.

Human pancreas biopsy specimens were selected from donor material made available from the Beta Cell Bank in Brussels, for studies approved by Medical Ethics Committee of the University Hospital UZ Brussel (BUN-B14320072705), that were in part previously published [14]. The samples were taken as part of a quality control procedure from organs processed for islet isolation and transplantation and can thus under the Belgian Law of 18 December 2008 be used for research. Donor pancreas was obtained from the Eurotransplant Foundation (Leiden).

Sections were double stained for Ki67 and insulin with streptavidin horseradish peroxidase or alkaline phosphatase complex (both from Dako) [14]. Insulin positive aggregates were scored for their number of beta cells as well as the number of Ki67-positive beta cells per aggregate, after counting on average 6000 beta cells per case.

### Statistical Analysis

Values are expressed as means with SD. Statistical significance was assessed by Student’s t test.

## Results

### Duct-ligation in Pancreatic Tails Leads to Measurement of Increased Beta Cell Mass in Absence of Increase in Total Beta Cell Number

**Figure 1:**
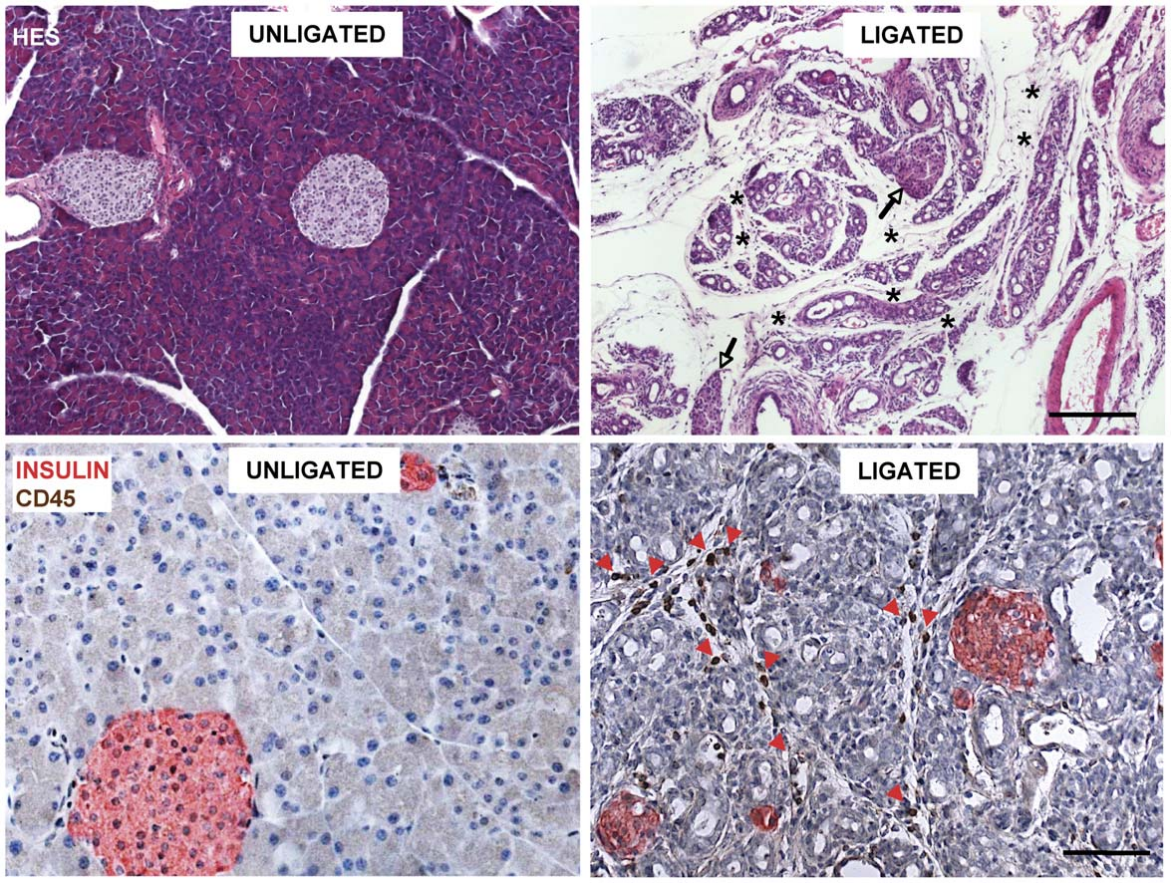
Comparison of tissue composition in unligated (left) and ligated (right) pancreatic tails. Two weeks after duct ligation the tissue is devoid of acini. It mainly consists of ducts with interspersed islets (arrows) and enlarged fibrous septa and adipose tissue (stars). It exhibits an inflammatory infiltration with numerous CD45-positive leucocytes in the septa (red arrowheads); these cells were virtually absent in unligated tissue. The degree of infiltration was less pronounced than after one week. Hematoxylin-eosin, scale bar 200 µm (upper panel) and immunohistochemistry for insulin (red) and CD45 (brown), scale bar 100 µm (lower panel).

Two weeks after ligation of pancreatic tails, their weight had decreased to 25 percent of that of unligated tails (Table 1). The lobes were devoid of acinar cells and now mainly consisted of ductal structures with interspersed islets; they were separated by enlarged fibrous septa and adipose tissue (Figure 1). An inflammatory cell infiltration was noticed, with numerous CD45-positive leucocytes in the septa (Figure 1); this infiltration was less pronounced than after one week, but clearly distinct from the state in sham tissue. Leucocytes do occur in unligated tissue, but they are so scarce that a “concentration” effect following acinar cell depletion can only partially explain their numbers in PDL-tails; moreover, their higher abundance at week 1 is consistent with an infiltration.

Quantification of beta cell mass in ligated tails indicated a 60 percent higher weight, which confirms previous reports [1], [2], [8], [9]; however, this was not accompanied by a rise in total beta cell number (Table 1). This discrepancy cannot be attributed to a larger individual beta cell size in PDL-tails (Table 1). Beta cell size was calculated by dividing total beta cell volume by the counted total cell number, assuming a spherical cell shape of the cells. This method is stereologically valid as it takes into account section thickness, both for measurement of total beta cell volume as for counting total beta cell number. Direct measurement of cell size in the sections led to the same conclusion, namely that the average beta cell size in PDL tails is not larger than in controls (151±16 µm^2^ versus 150±22 µm^2^). Whatever method used in tissue sections, the values obtained for cell size are likely to differ from those in fresh tissue, being influenced by tissue processing and shrinkage.

The higher total beta cell mass (weight) in PDL tails was neither associated with an increase in total beta cell volume (Table 1). This discrepancy appears caused by the tissue alterations after PDL, which have significantly enlarged the compartment of septa and adipocytes. This compartment contributes to the measured weights that serve to calculate the weight of the beta cell mass and will therefore lead to its overestimation [8]. It does however contribute markedly less to the total volumes as measured automatically by the Cavalieri method, where it is in part not recognized, being space without nuclei; consequently the beta cell volumes in PDL tails will not be overestimated as is the case for beta cell mass data. This explanation is supported by comparing total tail volumes measured by either the Archimedes or the Cavalieri principle: both methods generated similar values for sham tails, while the Cavalieri technique resulted in a 40 percent lower volume for PDL tails.

### Duct Ligation Induces Neoformation of Small Beta Cell Clusters with Increase in Associated Number of Beta Cells

Duct-ligated tails exhibited a 2-fold increase in the total number of beta cell aggregates (Table 1). This was almost exclusively the result of neoformation of small clusters (defined as a diameter of an insulin-positive area <50 µm, which corresponds to 1–13 sectioned beta cells), the number of which increased from 1.9 10^3^ to 4.2 10^3^. It was associated with a doubling of the beta cell number in this category; however, this increase was insufficient to significantly increase total beta cell numbers (Table 1). There was also a modest increase in the number of aggregates with diameter 50–100 µm and the associated beta cell number, but not in the category >100 µm which contains the majority of beta cells in both duct-ligated and sham tails (more than 80 percent of total) (Table 1).

The increase in beta cell number in aggregates <100 µm (together 17 000 more cells at week 2) was associated with a comparable increase in alpha cells (13 000 more cells) (Table 1). When compared to their respective baseline levels in sham tails (255 000 beta cells and 81 000 alpha cells), the increase in alpha cell number represents a higher fraction (16 percent) which explains the significance of its contribution to total alpha cell number (Table 1).

We have not determined to which extent the increase in beta cell number in the small size category includes a rise in the number that is located in the duct epithelium. This is anyway a small subpopulation. It nevertheless appeared that it was now also present in small ducts whereas we only found it in large ducts of sham tails. This was also the case for the glucagon-positive cells within the ducts.

### Duct Ligation Induces Proliferating Beta Cells in Small Beta Cell Clusters

**Figure 2:**
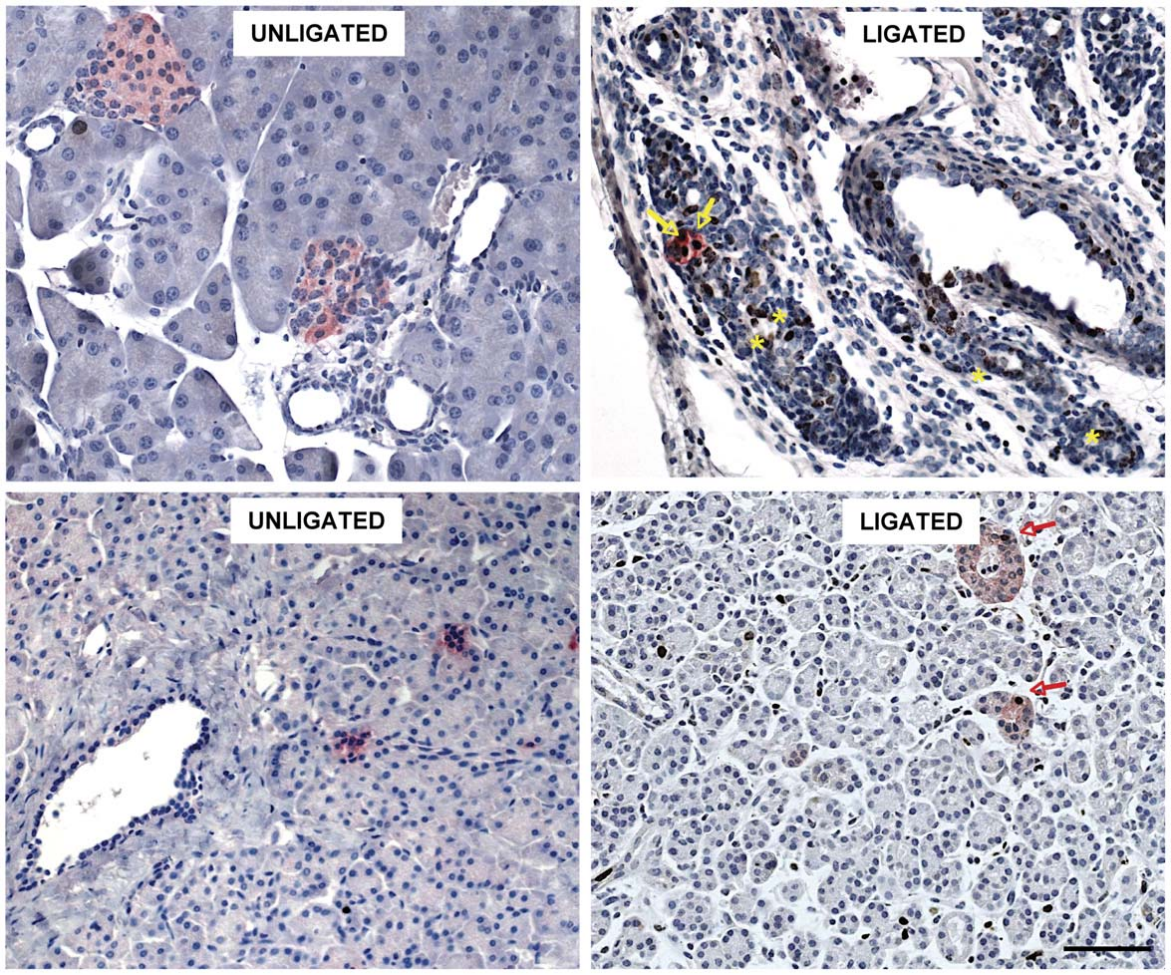
Proliferation-activated beta cells in small beta cell clusters of PDL-mouse tails (left panel) and of human pancreas with inflammatory infiltration (right panel). Two weeks after PDL pancreatic tails contain a larger pool of Ki67-positive beta cells in the small beta cell clusters (arrows in left panel) (391±84 cells versus 27±27 in unligated tails, p<0,01). They also present higher percentages of Ki67-positive cells in large and small ducts (arrowheads). A number of human donor organs were found to present a diffuse inflammatory infiltration [14]. The small beta cell clusters in three consecutively analysed organs contained higher percentages of Ki67-positive beta cells (arrows in right panel) than those in age- and gender-matched controls without infiltration (3, 0.8 and 1.4% versus ≤0.1%). Immunohistochemistry for insulin (red) and Ki67 (brown), scale bar 100 µm.

We examined whether the small beta cell clusters (<50 µm) in duct-ligated tails exhibited markers that may disclose potential progenitors. No ngn3-immunoreactive cells were detected, and neither were insulin-positive cells found that were also positive for cytokeratin or glucagon. On the other hand, this category of beta cells contained a significantly higher percent Ki67-positive cells (1.9±1.1 in ligated tails versus <0.2 percent in the sham group, p<0.01) (Figure 2). The ducts in the ligated tissue also contained significantly more Ki67-positive cells (Figure 2), none of which were found to express insulin. The increase in the number of proliferating beta cells in the fraction <50 µm (400 cells) significantly contributed to the increase in the pool of these cells in the entire tail (1800 cells): 22 percent, whereas its beta cell number represents only 8 percent of total.

### Proliferating Beta Cells in Small Beta Cell Clusters of the Human Pancreas

The observation that small beta cell clusters contribute a significant fraction of proliferating beta cells in mouse pancreases with inflammatory infiltrates led us to analyze the small beta cell clusters (<50 µm) in human donor pancreases with increased levels of inflammatory cells, including CD68+ macrophages and CD45+ leucocytes. These organs had been identified during routine analysis of donor organs [14]. They were found to contain higher percentages of Ki67-positive beta cells when compared with age-, BMI and gender-matched donor organs without infiltration [14]; increases were also seen in the percent Ki67-positive non-endocrine cells.

Three cases with a higher percent of Ki67-positive beta cells (>95% percentile) were selected and three matched controls. In each case we analyzed 300 to 1500 beta cells that were located in small beta cell clusters, i.e. 1 to 10 sectioned cells. In two controls we did not find Ki67-positive cells (<0.1%) whereas 0.1% was counted in one. In the three donors with an overall high percentage of these cells, the small beta cell clusters contained respectively 3, 0.8 and 1.4 percent Ki67-positive beta cells (Figure 2). Similar to the duct-ligated mouse tails, higher percentages of Ki67-positive cells were also found in the ducts. These data indicate that small beta cell clusters in the human pancreas can also significantly contribute to the pool of proliferating beta cells. A more extensive study on larger donor numbers will be needed to compare this contribution to that by the beta cells in large islets.

## Discussion

The pancreatic tail of 10-week-old mice was found to contain approximately 250×10^3^ beta cells which, on a body weight basis (10×10^6^ per kg), is 2-fold higher than in 10-week old rats [11]. Their distribution is comparable to that in rats: 85 percent is located in islets with diameter >100 µm - which represent only 6 percent of all beta cell aggregates - and 5 percent in clusters <50 µm - which account for more than 80 percent of all aggregates.

Two weeks after duct ligation, tails exhibited a 60 percent higher beta cell mass, which is consistent with the rise reported by previous studies [1], [2], [8], [9]. It was however not associated with an increase in total beta cell number. This discrepancy is not attributable to a larger average size of the beta cells in PDL tails. It appears caused by an overestimation of the weight of the beta cell compartment after PDL as a result of a markedly enlarged compartment of extracellular and adipose tissue that contributes to the weight of PDL tails. Our data confirm the concern of Kopp et al. on beta cell mass measurements in the PDL pancreas [8]. The PDL-induced tissue alterations, with a loss of acinar cells, hyperplasia of small duct cells, fatty degeneration, fibrosis and profound changes in interstitial space [1], [2], [8] indeed interfere with the assessment of the pancreatic surface area in the sections. This enlarged compartment does not interfere with the direct beta cell count, nor with the measurement of total beta cell volume through the Cavalieri method as this is not based on total pancreas surface area. The present data illustrate the advantage of quantification methods that count total beta cell numbers over indirect assessments using weight of the beta cell population [11]. Direct and indirect measurements will often correlate, in particular within the same experimental model, but this does not argue against the preference of counting beta cells when the purpose is to assess loss, growth or regeneration of beta cells. In addition, indirect methods can be influenced by other factors than beta cell number, as in the case of PDL. These facts and considerations can explain differences in published values for beta cell mass and volume [1], [2], [4], [8], [9], [15], [16]. Data combined from different laboratories certainly allow overall plots on postnatal growth curves [17] but will most probably not be sensitive enough to quantify influences within this process or more subtle changes.

When beta cell numbers in PDL-tails were analyzed per size category of their aggregates, a 95 percent increase was measured in the small cluster category (<50 µm). It resulted from a doubling in the number of these clusters, and not from an increase in their average beta cell number. Since this doubling of small clusters is not associated with a decrease in the number of large aggregates, it can be attributed to their neogenesis instead of their formation from dispersed large aggregates. This PDL-induced neogenesis of small clusters was associated with an activation of beta cell proliferation in this category (1.9 percent Ki67-positive beta cells versus <0.2 percent in sham), which led to a 12-fold increase in their number of proliferating beta cells. This might account for a growth of a number of these particles to larger sizes, as indicated by higher particle numbers with diameters 50–100 µm, and associated higher numbers of beta cells in this fraction. PDL also increased the pool of proliferating beta cells in the larger size categories but to a lesser extent (2-fold). Alpha cells were found associated to the small beta cell clusters; their number increased in parallel, to the same magnitude as that of beta cells. The neoformed beta cells in this small cluster fraction represented only a minor proportion (<5%) of total beta cell numbers, which can explain the lack of a statistically significant increase in the latter; even in case of statistical significance, the magnitude is very small. We have not examined whether the neogenesis of small endocrine clusters and growth to larger aggregates proceeds beyond week 2, possibly leading to increased total beta cell numbers at later time points.

In the human pancreas, a higher percent of proliferation-activated beta cells was recently reported in conditions with inflammatory infiltrates consisting of macrophages and CD45-positive leucocytes [14]; there were also higher percentages of proliferating duct cells. When re-analyzing these cases we observed that the small beta cell clusters participated to this proliferation activation.

Previous studies have reported that PDL induces neogenesis of beta cells as well as a beta cell proliferative activity [1], [2]. The source for neogenesis is still under debate. In rats, PDL was found to transiently increase the relative proportion of small beta cell clusters containing CK20-insulin double positive cells, which was taken as suggestive evidence for a ductal origin [2]. Subsequent lineage tracing studies in adult mice led to conflicting conclusions on the formation of beta cells from duct cells following PDL in adult mice [3], [8], [9], [18]. Neogenesis through transdifferentiation of acinar cells [5] and of alpha cells [4] has also been proposed. The identification of ngn3-expressing progenitor cells [1] may point to the existence of a stem cell or earlier progenitors, but have also been interpreted as early stage islet cells [8]. Virtually all these studies sought a relationship between a neogenic process and the increase in beta cell mass, measured after PDL, considered to express a regenerative response leading to increased total beta cell numbers. Our data have raised concerns for doing this. First, the neogenesis of small clusters is in itself not sufficient to increase total beta cell number, and can also occur in absence of such increase. It seems therefore more relevant to count numbers in, and of, small clusters, the sites where neogenesis occurs. Second, measurements of beta cell mass overestimate the size of the beta cell population after PDL, another argument for using methods that count beta cell numbers.

In conclusion, duct ligation causes tissue alterations that make beta cell mass measurements inadequate as quantification method in this model. Direct counts of the number of beta cells and their distribution over beta cell aggregates of different size have demonstrated that neogenesis of small beta cell clusters can be induced in the normal adult pancreas; the associated increase in beta cell number is however insufficient to elevate total beta cell number; on the other hand, the associated increase in the number of alpha cells in small clusters significantly increased total alpha cell numbers. The PDL-induced neogenesis of small clusters was associated with a 12-fold increase in their pool of proliferating beta cells. It is conceivable that this growth process at the level of small beta cell clusters is caused by the inflammatory infiltration that characterizes the PDL-tails. Interestingly, human pancreases with a similar infiltration by macrophages and CD45-cells also exhibited small beta cell clusters with more proliferation-activated beta cells. In fact, normal mouse and human pancreas exhibit very low percentages of Ki67-positive beta cells in their small clusters. We previously noticed that beta cells in small clusters of the normal adult rat pancreas are well granulated and remained so following a glibenclamide-treatment that degranulated beta cells in the larger clusters [11]. Although our study does not define the role of small beta cell clusters, it identifies them as a distinct component in the functional heterogeneity of the pancreatic beta cell population. Their major function may not be related to insulin secretion but to growth of the beta cell population involving [1] their neogenesis with proliferation activation of their beta cells, and [2] their fusion and/or growth to larger islets with beta cell functions that are needed for metabolic control. This second component was not observed in the present study conducted on non-diabetic animals. Further studies should investigate how this second component can be induced, possibly requiring models with reduced beta cell numbers at start.

## References

[1] Xu X, D’Hoker J, Stangé G, Bonné S, De Leu N, et al (2008) β Cells Can Be Generated from Endogenous Progenitors in Injured Adult Mouse Pancreas. Cell 132: 197–207. 18243096 10.1016/j.cell.2007.12.015

[2] Wang RN, Kloppel G, Bouwens L (1995) Duct- to islet-cell differentiation and islet growth in the pancreas of duct-ligated adult rats. Diabetologia 38: 1405–1411. 8786013 10.1007/BF00400600

[3] Inada A, Nienaber C, Katsuta H, Fujitani Y, Levine J, et al (2008) Carbonic anhydrase II-positive pancreatic cells are progenitors for both endocrine and exocrine pancreas after birth. Proc Natl Acad Sci U S A 105: 19915–19919. 19052237 10.1073/pnas.0805803105 PMC2604974

[4] Chung CH, Hao E, Piran R, Keinan E, Levine F (2010) Pancreatic beta-Cell Neogenesis by Direct Conversion from Mature alpha-Cells. Stem Cells 28: 1630–1638. 20653050 10.1002/stem.482

[5] Bertelli E, Bendayan M (1997) Intermediate endocrine-acinar pancreatic cells in duct ligation conditions. Am J Physiol 273: C1641–1649. 9374650 10.1152/ajpcell.1997.273.5.C1641

[6] Criscimanna A, Speicher JA, Houshmand G, Shiota C, Prasadan K, et al. (2011) Duct Cells Contribute to Regeneration of Endocrine and Acinar Cells Following Pancreatic Damage in Adult Mice. Gastroenterology. 10.1053/j.gastro.2011.07.003 PMC4326039 21763240

[7] Nakamura K, Minami K, Tamura K, Iemoto K, Miki T, et al (2011) Pancreatic beta-cells are generated by neogenesis from non-beta-cells after birth. Biomed Res 32: 167–174. 21551953 10.2220/biomedres.32.167

[8] Kopp JL, Dubois CL, Schaffer AE, Hao E, Shih HP, et al (2011) Sox9+ ductal cells are multipotent progenitors throughout development but do not produce new endocrine cells in the normal or injured adult pancreas. Development 138: 653–665. 21266405 10.1242/dev.056499 PMC3026412

[9] Solar M, Cardalda C, Houbracken I, Martin M, Maestro MA, et al (2009) Pancreatic exocrine duct cells give rise to insulin-producing beta cells during embryogenesis but not after birth. Dev Cell 17: 849–860. 20059954 10.1016/j.devcel.2009.11.003

[10] Dor Y, Brown J, Martinez OI, Melton DA (2004) Adult pancreatic beta-cells are formed by self-duplication rather than stem-cell differentiation. Nature 429: 41–46. 15129273 10.1038/nature02520

[11] Chintinne M, Stangé G, Denys B, In ‘t Veld P, Hellemans K, et al (2010) Contribution of postnatally formed small beta cell aggregates to functional beta cell mass in adult rat pancreas. Diabetologia 53: 2380–2388. 20645074 10.1007/s00125-010-1851-4

[12] Gundersen HJ, Jensen EB, Kieu K, Nielsen J (1999) The efficiency of systematic sampling in stereology–reconsidered. J Microsc 193: 199–211. 10348656 10.1046/j.1365-2818.1999.00457.x

[13] Svenstrup K, Skau M, Pakkenberg B, Buschard K, Bock T (2002) Postnatal development of beta-cells in rats. Proposed explanatory model. Apmis 110: 372–378. 12076254 10.1034/j.1600-0463.2002.100502.x

[14] In’t Veld P, De Munck N, Van Belle K, Buelens N, Ling Z, et al (2010) Beta-cell replication is increased in donor organs from young patients after prolonged life support. Diabetes 59: 1702–1708. 20413508 10.2337/db09-1698 PMC2889770

[15] Hakonen E, Ustinov J, Mathijs I, Palgi J, Bouwens L, et al. (2011) Epidermal growth factor (EGF)-receptor signalling is needed for murine beta cell mass expansion in response to high-fat diet and pregnancy but not after pancreatic duct ligation. Diabetologia. 10.1007/s00125-011-2153-1 21509441

[16] Rooman I, Lardon J, Bouwens L (2002) Gastrin stimulates beta-cell neogenesis and increases islet mass from transdifferentiated but not from normal exocrine pancreas tissue. Diabetes 51: 686–690. 11872667 10.2337/diabetes.51.3.686

[17] Bonner-Weir S (2000) Islet growth and development in the adult. J Mol Endocrinol 24: 297–302. 10828822 10.1677/jme.0.0240297

[18] Kopinke D, Brailsford M, Shea JE, Leavitt R, Scaife CL, et al (2011) Lineage tracing reveals the dynamic contribution of Hes1+ cells to the developing and adult pancreas. Development 138: 431–441. 21205788 10.1242/dev.053843 PMC3014632

